# Predominance of Uganda genotype of *Mycobacterium tuberculosis* isolated from Ugandan patients with tuberculous lymphadenitis

**DOI:** 10.1186/s13104-015-1362-y

**Published:** 2015-09-01

**Authors:** Dan Wamala, Moses Okee, Edgar Kigozi, David Couvin, Nalin Rastogi, Moses Joloba, Gunilla Kallenius

**Affiliations:** Department of Pathology, Mulago Hospital and Makerere University College of Health Sciences, P. O. Box 7072, Kampala, Uganda; Department of Medical Micobiology, Makerere University College of Health Sciences, P. O. Box 7072, Kampala, Uganda; Unité de la Tuberculose et des Mycobactéries, Institut Pasteur de Guadeloupe, Pointe-à-Pitre, Guadeloupe France; Department of Clinical Sciences and Education, Sodersjukhuset. Karolinska Institute, 171 77 Stockholm, Sweden

## Abstract

**Background:**

In Uganda, the emerging Uganda genotype of *Mycobacterium tuberculosis* is the most common cause of pulmonary tuberculosis (PTB), and accounts for up to 70 % of isolates. Extrapulmonary TB (EPTB) is less studied in Uganda.

**Methods:**

Molecular characterization using deletion analysis and spoligotyping was performed on 121 *M*. *tuberculosis* isolates from lymph node fine needle biopsy aspirates of Ugandan patients with tuberculous lymphadenitis. The evolutionary relationships and worldwide distribution of the spoligotypes were analyzed.

**Results:**

*Mycobacterium tuberculosis* was the only cause of EPTB in this study. The T2 sublineage was the most predominant lineage and the Uganda genotype was the dominant genotype. There were 54 spoligotype patterns among the 121 study isolates. The dominant spoligotypes were shared international types (SIT) SIT420, SIT53, SIT 135, SIT 128 and SIT590 in descending order. All but SIT420 were previously reported in pulmonary TB in this setting. The phylogenetic analysis showed a long descendant branch of spoligotypes belonging to the T2-Uganda sublineage containing specifically SITs 135, 128 and 420.

**Conclusion:**

In most cases, the spoligotypes were similar to those causing PTB, but the Uganda genotype was found to be less common in EPTB than previously reported for PTB in Uganda. The phylogenetic analysis and the study of the worldwide distribution of clustered spoligotypes indicate an ongoing evolution of the Uganda genotype, with the country of Uganda at the center of this evolution.

**Electronic supplementary material:**

The online version of this article (doi:10.1186/s13104-015-1362-y) contains supplementary material, which is available to authorized users.

## Background

Tuberculosis (TB) is a leading infectious cause of morbidity and mortality, responsible for an estimated annual 8.2 million new cases and 1.4 million deaths globally [1]. About one quarter of TB cases occur in Africa [1]. Uganda with an incidence of 299 and a mortality of 84 cases per 100,000 per year, is ranked 16th among the 22 countries with the heaviest burden of TB [2]. Although pulmonary TB (PTB) is the major type of TB, extrapulmonary TB (EPTB) [3] is associated with a high morbidity and mortality [4] and requires special attention [5]. TB lymphadenitis is the commonest extrapulmonary manifestation of EPTB [6]. Of 4072 patients seen in 2012 at the Mulago National Referral Hospital (MNRH) Tuberculosis Clinic, the largest TB treatment center in Uganda, 902 (22 %) had EPTB [7]. EPTB also offers diagnostic challenges because of the paucibacillary nature of EPTB specimens, necessitating culture and molecular analysis for efficient diagnosis.

*Mycobacterium tuberculosis* has evolved into genetically diverse lineages and sublineages [8], and the genetic variety has ramifications including significant variation in virulence [9]. The development of molecular techniques allows for the identification and tracking of individual strains of *M*. *tuberculosis* [10]. In Uganda, the emerging Uganda genotype of *M*. *tuberculosis* is the prevalent cause of PTB, and accounts for up to 70 % of isolates [11]. In a recent study of tuberculous lymphadenitis patients, we found that the Uganda genotype was also a major aetiological agent in EPTB [12].

Our objective was to analyze the genetic diversity of the clinical isolates from this study [12], and to compare them with the genotypic patterns of PTB isolates reported previously in this setting [11] in order to understand the epidemiological and phenotypic characteristics of *M*. *tuberculosis* strains causing EPTB.

## Methods

### Ethical considerations

The study was reviewed and approved by the Institutional Review Board of Makerere University School of Medicine and the Uganda National Council for Science and Technology. Written informed consent to obtain samples as well as to use isolates from the samples for studies was obtained from all enrolled study participants or their legal guardians.

### Study design and population

Isolates of *M*. *tuberculosis* complex were studied from patients presenting with superficial persistent lymph node enlargement, at the Fine Needle Aspiration Clinic, at MNRH, Kampala. Altogether 121 lymph nodes isolates, each representing one patient, collected between February 2010 and July 2012 were included. EPTB patients were included with or without pulmonary clinical features.

The patients’ clinical, radiological and pathological features were described previously [12]. Of the patients in the study, 58 (47.9 %) were males with a mean age of 30.3 years and 62 (52.1 %) were females with a mean age of 27.1 years. The gender of one patient was not recorded. Seventy-five (65.8 %) were HIV seropositive while 39 (34.2 %) were HIV negative. Seven (5.9 %) did not consent to HIV testing.

To confirm that all the isolates were mycobacteria, polymerase chain reaction (PCR) was performed using 16s reverse and 16s forward primers (Integrated DNA Technologies) to target the *16s rRNA* region with a conserved sequence that is typical for genus *Mycobacterium* [13–15].

Furthermore, using an in-house PCR, we confirmed presence of *M*. *tuberculosis* complex members by amplification of the insertion sequence IS*6110* with aid of reverse and forward IS*6110* primers (Integrated DNA Technologies). Presence of bands on the gel of a size of approximately 500 bp signified positive results [16].

### Region of difference analyses

The lineages and sublineages of *M*. *tuberculosis* isolates were determined by PCR based genomic deletion analysis for the presence or absence of specific regions of difference (RD), RD1, RD4, RD9, RD14, RD724, RD750 and RD105 using standard protocol [13]. RD 9 ruled out other species and confirmed that the cases were *M*. *tuberculosis*, RD4 and RD 14 ruled out *M. bovis*, the RD724 deletion is characteristic of Uganda genotype while RD105 deletion is specific for Beijing strains. The analyses were performed using a previously described method [13].

### Spoligotyping

All *M*. *tuberculosis* complex strains were assayed by spoligotyping using standard protocols [10] and following manufacturer’s instructions (reagents from Ocimum Biosolution, custom MasterMix from ABgene). The presence of spacers was visualized on film as black squares after incubation with streptavidin-peroxidase and ECL chemiluminescence detection reagents (RPN 2105 Amersham, GE Healthcare Bio-sciences). The spacer hybridization patterns were translated into binary and octal format as previously described [17]. The 43-digit binary code was converted to 15-digit octal code (base 8, having the digits 0–7) [17].

The binary codes of the isolates were entered into the SITVIT2 database of the Pasteur Institute of Guadeloupe and assigned specific shared international spoligotype signatures (SIT) according to the SITVIT2 data base [18].

### Identification of Uganda genotype

The Uganda genotype is a sublineage of the T2 lineage and was identified by the lack of RD724 on RD analysis [19], and by the lack of spacers 33–36 and spacer 40 and/or 43 by spoligotyping [20, 21].

### Phylogenetic analysis

The evolutionary relationships between the spoligotypes of the 121 isolates was studied by (a) a Minimum Spanning Tree (MST) constructed on all isolates, (b) a spoligoforest tree drawn as a hierarchical layout and (c) a spoligoforest tree drawn using the Fruchterman–Reingold algorithm. Both spoligoforests were drawn using the spolTools software (http://www.emi.unsw.edu.au/spoltools/) [22, 23].

The worldwide distribution of clustered spoligotypes found in this study was further investigated using the SITVIT2 database, and was recorded for regions and countries representing ≥3 % of a given SIT as compared to their total number in the SITVIT2 database. The various macro-geographical regions and sub-regions were defined according to the specifications of the United Nations (http://en.wikipedia.org/wiki/ISO_3166-1_alpha-3).

### Data analysis

Clinical-demographic data was entered into the computer using Epidata 3.1 software and then exported to SPSS version 21 for analysis. The spoligotyping data were digitized and analyzed with the BioNumerics software, version 5.0 (Applied Maths, Kortrijk, Belgium).

Statistical association between the dominant strain types and HIV sero-status were analyzed in a 2 × 2 contingency table using Fisher’s exact test. A p value of <0.05 was considered statistically significant. The independent T test was used to determine whether there was a statistically significant difference between the female and male mean ages.

## Results

### Genus and species identification

All of the 121 isolates were confirmed to be *M*. *tuberculosis* complex based on 16S-rRNA as well as the presence of the insertion sequence IS*6110* that is highly conserved in all members of *M*. *tuberculosis* complex.

### Spoligotypes

All of the 121 isolates had spoligotypes characteristic of *M*. *tuberculosis*. The spoligotype octal code outcome results were compared to the SIT and lineages/sublineages described in the SITVIT2 database. Spoligotyping revealed 52 distinct spoligotype patterns, of which 32 patterns matched a pre-existing SIT in the SITVIT2 database, whereas 4 SITs were newly created either within the present study or after a match with an orphan in the database (Table 1). Sixteen patterns were true orphans (Additional file 1: Table S1).

**Table 1:**
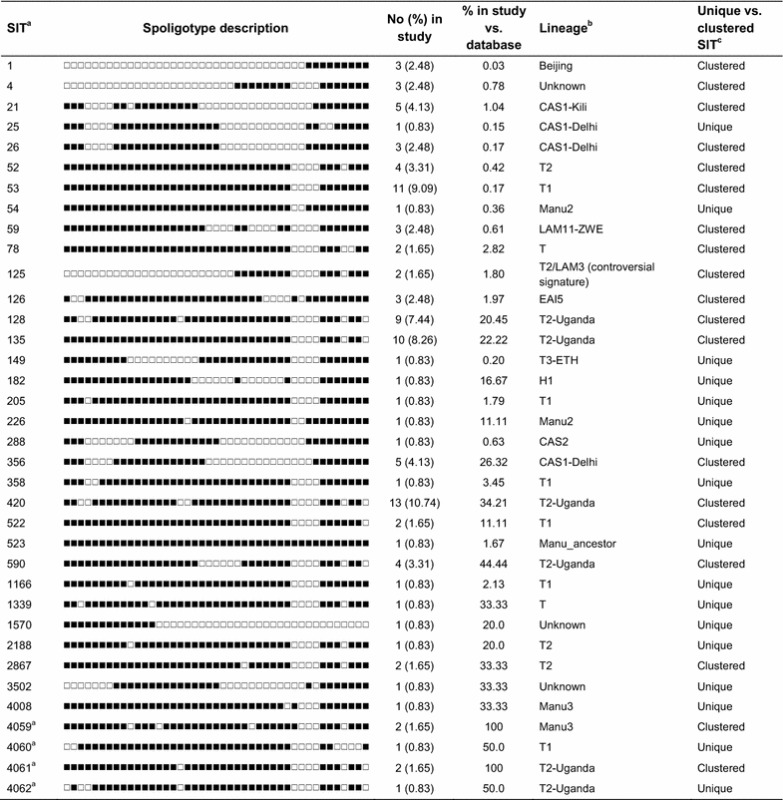
Description of 36 SITs and corresponding spoligotyping defined lineages/sublineages starting from a total of 121 *M*. *tuberculosis* strains isolated in Kampala, Uganda

^a^Newly created SITs are marked by lowercase alphabet a. Country distribution for newly created SITs was as follows: SIT4059^a^, n = 2 this study; SIT4060^a^, n = 1 this study, n = 1 PRT; SIT4061^a^ n = 2 this study; SIT4062^a^ n = 1 this study, n = 1 ZMB

^b^Lineage designations according to SITVIT2; “unknown” designates patterns with signatures that do not belong to any of the major lineages described in the database

^c^Clustered strains correspond to a similar spoligotype pattern shared by 2 or more strains “within this study”; as opposed to unique strains harboring a spoligotype pattern that does not match with another strain from this study. Unique strains matching a preexisting pattern in the SITVIT2 database are classified as SITs, whereas in case of no match, they are designated as “orphan”

The T2 sublineage was predominant comprising 55 (46 %) isolates, of which 46 were T2-Uganda, characterized by the absence of spacers 33–36 and spacer 40 and 43 [24], while 2 isolates had the controversial signature T2/LAM3. The T2 sublineage was followed in frequency by the T1 sublineage with 19 (16 %) isolates. The Central Asian Strain (CAS) family comprised 16 (18 %) isolates, of which CAS1-Dehli was the most common with 10 isolates followed by CAS1-Kili with 5 isolates. Other sublineages were EAI5 with 3 isolates, Beijing 3, LAM11-ZWE 3, MANU3 3 and LAM1 2. Seven spoligotypes could not be assigned to any sublineage.

The two predominant spoligotypes in our sample were SIT420 (T2-Uganda) with 13 (10.7 %) and SIT53 (T1) with 11 (19.8 %) isolates. Other significant spoligotypes identified in the sample were SIT128 (T2-Uganda) with 9 (7.4 %) and SIT135 (T2-Uganda) with 10 (8.3 %) isolates (Table 1). Sixteen isolates had no assigned SIT according to the SITVIT2 database and were true orphans (Additional file 1: Table S1). None of the predominant spoligotypes SIT420, SIT53 and SIT135 was significantly associated with HIV infection. No association was found between the major spoligotypes and HIV infection (Additional file 2: Table S2).

Of the 12 SIT420 patients tested for HIV, 8/74 (10.8 %) were HIV negative whereas 4/38 (10.5 %) were HIV positive (p = 1.00). One patient did not consent to HIV testing. Of ten SIT135 patients tested for HIV, 6/74 (8.1 %) were HIV negative while 4/38 (10.5 %) were HIV positive (p = 0.732). Ten SIT53 patients tested for HIV, 9/74 (12.2 %) were HIV negative and 1/38 (2.6 %) were HIV positive (p = 0.16). One SIT53 patient did not consent to HIV testing.

No correlation between a particular spoligotype and gender or age was found (Additional file 3: Table S3).

### RD analysis

RD9 analysis confirmed that all isolates were *M*. *tuberculosis*, while RD4 and RD14 analysis ruled out *M. bovis*. RD105 analysis identified three Beijing isolates. RD724 analysis revealed 55 (46 %) isolates to be Uganda genotypes, that is all the 55 isolates with a T2 spoligotype signature, lacking spacers 33–36 and spacer 40 and/or 43.

Two of the isolates, designated SIT125, which were deleted for RD724, were initially assigned to the LAM lineage according to the SITVITWEB database, although the spoligotype signature was a typical T2. Their designation in the SITVIT2 database has now been changed to T2/LAM3.

### Phylogenetic analysis

**Fig. 1:**
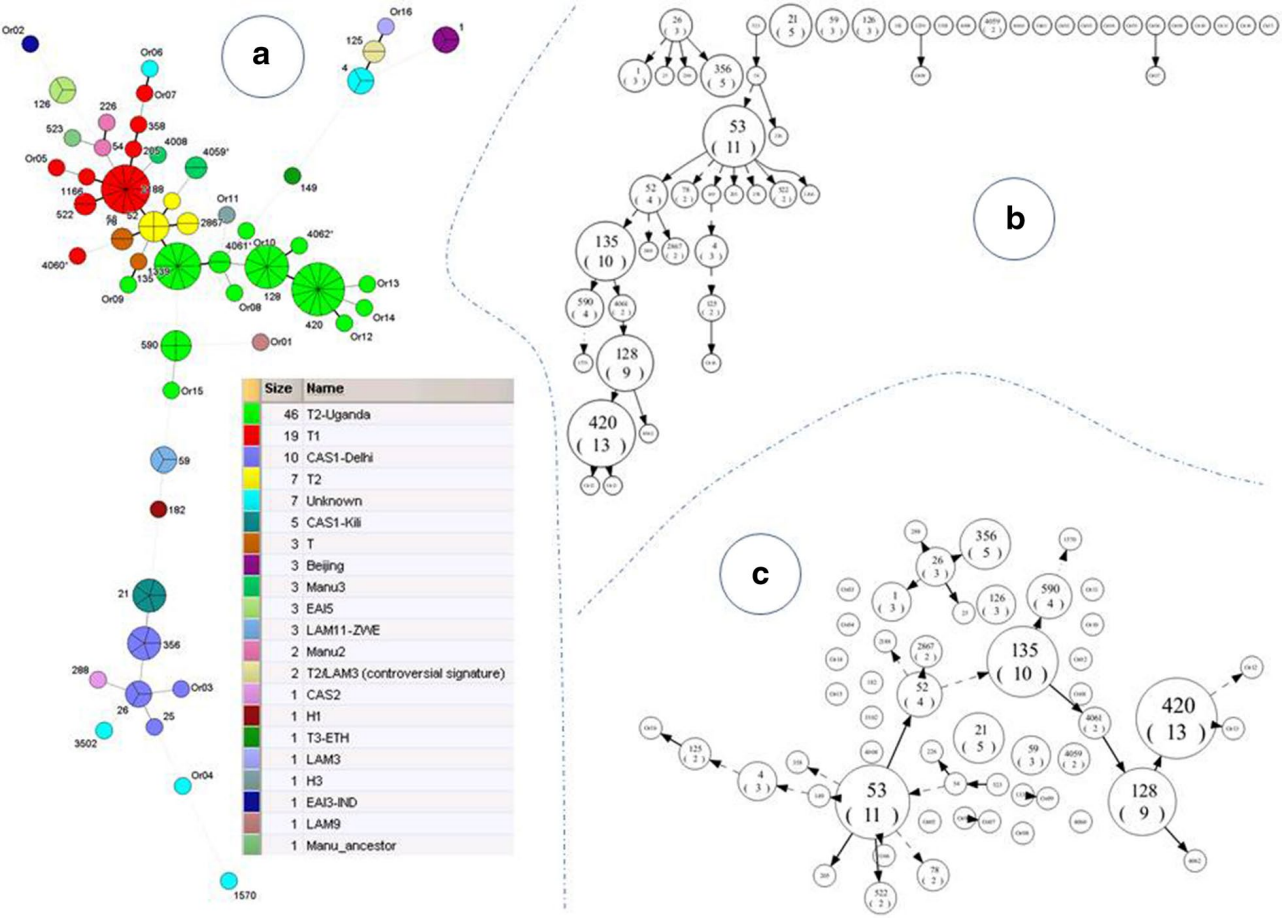
Phylogenetical analysis illustrating evolutionary relationships between *M*. *tuberculosis* spoligotypes in Kampala, Uganda (n = 121 isolates). **a** Minimum Spanning Tree (MST) constructed on all isolates. *Separations between the nodes* represent the number of strains shared by a given spoligotype pattern. The *links between nodes* indicate the distance (*darker and bolder lines* mean a unique change whereas *finer gray lines*, *continued*, *dotted* or *dashed*, indicate more changes). **b** Spoligoforest tree drawn as a hierarchical layout; and **c** spoligoforest tree drawn using the Fruchterman–Reingold algorithm. Both spoligoforests were drawn using the spolTools software (http://www.emi.unsw.edu.au/spoltools/). Loss of spacers is represented by *directed edges* between nodes, and the *arrowheads* point to descendant spoligotypes. The heuristic used selects a single inbound edge with a maximum weight using a Zipf model. *Solid black lines* link patterns that are very similar, i.e. loss of one spacer only (maximum weight being 1.0), while *dashed lines* represent links of weight comprised between 0.5 and 1, and *dotted lines* a weight less than 0.5

Phylogenetic analysis showed the evolutionary relationships between the spoligotypes of the 121 isolates (Fig. 1). One may notice in Fig. 1b, a long descendant branch of spoligotypes belonging to T2-Uganda sublineage containing specifically SITs 135, 128 and 420, which supports an ongoing evolution of this sublineage in Uganda.

### Worldwide distribution

We also attempted to describe the worldwide distribution of predominant SITs encountered in this study. As shown in Table 2, we observed that the isolates of the Uganda genotype were less frequently observed in countries outside Uganda (0–9 countries) than isolates of other sublineages (6–13 countries).

**Table 2:**
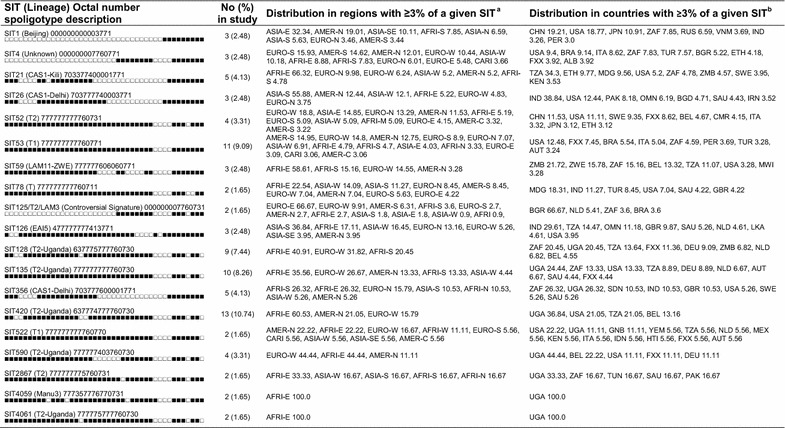
Description of clusters observed in our study and their worldwide distribution in the SITVIT2 database

^a^Worldwide distribution is reported for regions with more than 3 % of a given SITs as compared to their total number in the SITVIT2 database. The definition of macro-geographical regions and sub-regions (http://unstats.un.org/unsd/methods/m49/m49regin.htm) is according to the United Nations; Regions: AFRI (Africa), AMER (Americas), ASIA (Asia), EURO (Europe), and OCE (Oceania), subdivided in: E (Eastern), M (Middle), C (Central), N (Northern), S (Southern), SE (South-Eastern), and W (Western). Furthermore, CARIB (Caribbean) belongs to Americas, while Oceania is subdivided in four sub-regions, AUST (Australasia), MEL (Melanesia), MIC (Micronesia), and POLY (Polynesia). Note that in our classification scheme, Russia has been attributed a new sub-region by itself (Northern Asia) instead of including it among rest of the Eastern Europe. It reflects its geographical localization as well as due to the similarity of specific TB genotypes circulating in Russia (a majority of Beijing genotypes) with those prevalent in Central, Eastern and South-Eastern Asia

^b^The three letter country codes are according to http://en.wikipedia.org/wiki/ISO_3166-1_alpha-3; countrywide distribution is only shown for SITs with ≥3 % of a given SITs as compared to their total number in the SITVIT2 database. Note that FXX code designates Metropolitan France

## Discussion

In this study of the genotypic diversity of *M*. *tuberculosis* strains causing EPTB in Uganda, EPTB was found to be caused exclusively by *M*. *tuberculosis*. The Uganda genotype was the predominant genotype comprising 46 % of the isolates.

The Uganda genotype is defined by the RD724 deletion in combination with the lack of spacers 33–36 and spacer 40 and/or 43 by spoligotyping [20, 21]. Interestingly all isolates of the T2 sublineage were of the Uganda genotype, indicating that the RD724 deletion may be a marker of a majority of the T2 sublineage, a matter that needs to be further explored.

In a phylogenetic analysis SIT420 (T-2 Uganda), the most common spoligotype in this study was at the tip of a long descendant branch of spoligotypes belonging to the T2-Uganda sublineage, and this supports the ongoing evolution of this sublineage in Uganda. The fact that SIT420 is only reported in three other countries (USA, Tanzania and Belgium) in the SITVIT2 database supports the theory that Uganda is at the center of this evolution.

Following SIT420, SIT53 was the most prevalent spoligotype. SIT53 has been reported in several countries in sub-Saharan Africa. In a study performed in Cote d´Ivoire, SIT53 was the most predominant spoligotype in PTB retreated patients [25]. In a study from South Africa, SIT53 was the most frequent spoligotype and was associated with mixed infections [26]. However, in three previous studies in Uganda SIT53 was either rare [27, 28] or absent [11].

Two studies involved PTB patients from Southwestern Uganda [28] and peri-urban Kampala [11], respectively, while in one study the patients had either PTB or EPTB [27].

Although the Uganda genotype is the predominant genotype, it appears to be a less frequent aetiological agent in EPTB than in PTB. In a study of *M*. *tuberculosis* complex isolates from PTB done in the same setting [11], the Uganda genotype accounted for 70 % of isolates compared to 46 % seen in this current study. A more recent longitudinal study of sputum isolates in central Uganda also showed predominance of the Uganda genotype of *M*. *tuberculosis* at 63 % over a period from 1992 to 2009 [29], and in a study of sputum isolates done in Southwestern Uganda, 59.2 % of the isolates were of Uganda genotype [19]. In another study done on PTB and EPTB subjects in a rural central Uganda district hospital, 23 different spoligotypes were detected comprising predominantly T2 with subtypes Uganda I and Uganda II [27], which are both characterized by lack of hybridization to spacer 40, while in addition, strains of genotype Uganda I also lack spacer 43 [19].

The combination of spoligotyping and deletion analysis revealed one incongruence: two isolates, designated SIT125 (signature being loss of spacers 1–24, 33–36 and 40) which were deleted for RD724, were originally assigned to the LAM lineage according to the SITVIT2 database. By spoligotyping, the Euro-American lineage consists of the sublineages T, Haarlem, LAM, S and X. The spoligotype patterns of isolates from different lineages may converge by deletion of single or contiguous spacers in the direct repeat (DR) locus [20]. Although the phenomenon of homoplasy in the DR locus leading to convergent evolution to identical spoligotypes is considered a rare event [21], it argues against the use of spoligotyping for establishing phylogenetic relationships within the Euro-American lineage [30].

Indeed SIT125 with its characteristically ‘abridged’ profile could be linked either to T2 or LAM3 as well as an underlying S-lineage signature. It was initially classified as T2 in the SpolDB4 database and later reclassified as LAM3 in SITVITWEB, both being feasible based strictly on spoligotyping signatures. Although its definitive classification remains controversial, parallel data show its high phylogeographical specificity for Bulgaria [31]. In that paper, a possible relatedness with the SIT34 spoligotype (prototype of the S family) was suggested. Furthermore, an absence of LAM-specific IS*6110* insertion, which suggested that SIT125 (at least those from Bulgaria) were most probably not LAM. However, a study from Brazil on RDRio [32] showed that although 4/5 SIT125 strains were not LAM, 1/5 strain was LAM/RD-Rio. The SIT125 designation in the SITVIT2 database has now been changed to the controversial signature of T2/LAM3, while the exact phylogenetic position of SIT125 is debated and possible homoplasy in spoligotyping defined lineages is considered. For this reason, in this current study we have chosen to assign the two SIT125 isolates to the T2 lineage.

A previous PTB study from Uganda [11] indicated SIT128, SIT135, SIT52, SIT590 and SIT125 as the major SITs. SIT420, the predominant spoligotype in our study, was not identified in an urban area of Kampala [11] and was found only in low proportion in Southwestern Uganda [19] in patients with PTB. However, in a study from central Uganda [27] of both PTB and EPTB (lymph node isolates) SIT420 was the most prevalent with a cluster rate of 14.8 %, and mostly recovered from rural residents. In a study of sputum isolates from the neighboring country of Rwanda, SIT420 was the third most common spoligotype [33].

SIT52 constituted 3.3 % of the isolates compared to 7.6 % (26/334) seen in PTB in central Uganda [11] and 4.8 % (6/125) in Southwestern Uganda [19]. In Ethiopia, SIT54, SIT53 and SIT149 were the most dominant spoligotypes in EPTB while T1 was the most dominant sublineage [34]. Previous evidence from Italy however indicates that CAS lineages are associated with EPTB [35]. A more recent study of EPTB conducted in India also showed spoligotypes belonging to the CAS family (57.27 %) were predominant [36].

There is evidence of genotypic linkage of strains isolated from patients from a distinctive geographical region regardless of whether the patients acquired the infection from their current locality or had reactivated disease from their native country, this suggests that *M*. *tuberculosis* lineages may be adapted to particular genetic, cultural or environmental characteristics of the host [37]. Most of the genotypes in this study were similar to those previously found in PTB isolates in the same area [11], supporting the concept that EPTB and PTB are caused by the same type of strains and that mainly host factors determine whether PTB will transient through the lung to establish as chronic lymphatic disease [38]. The quantitative differences in spoligotype patterns could partly be due to the fact that Mulago Hospital is a national referral hospital, which has the largest TB diagnostic and treatment facilities in the country.

The lower prevalence of the Uganda genotype in this current study of EPTB isolates, compared to studies of PTB isolates in the same location indicates that other genotypes may be more prone to cause EPTB. The fact that the predominant SIT420 and SIT53 in this study was not seen in the PTB isolates previously studied [11] is another intriguing difference between EPTB and PTB in this setting. The observations merit future prospective studies in which isolates from patients from the same rural and urban region with EPTB and PTB are compared head to head at the same time.

Evidence from previous studies indicate that pathogen characteristics also determine whether patients present with PTB or EPTB [39, 40]. Thus, strains of the Euro-American lineage (to which the Uganda genotype belongs) may be more likely to cause pulmonary disease and less capable of extrapulmonary dissemination [41].Studies in the United States indicate that Indo-Oceanic and East Indian lineages of MTB were associated with a higher frequency of exclusively EPTB [42]. It is noteworthy that none of the predominant spoligotypes or sublineages was significantly associated with HIV infection.

In Uganda *M*. *bovis* control efforts are unsatisfactory which has increased the incidence of this infection in the population, and therefore the complete absence of *M*. *bovis* in the current study is worth noting. This absence of *M*. *bovis* was also seen in studies from Ethiopia [43]. Even in pastoralists [43] no *M*. *bovis* was isolated from lymph node fine needle biopsies despite close contact between humans and livestock, including consumption of raw milk and meat. But in another study from Southern Ethiopia, six of 35 (17.1 %) PCR positive patients with tuberculous lymphadenitis were found to have *M*. *bovis* [44]. Findings from a study in Tanzania of cervical adenitis patients confirmed that 10.8 % of the cases were due to *M*. *bovis* [45]. Thus, there is increasing evidence that *M*. *bovis* is at least a minor cause of human lymphadenitis in African countries.

## Conclusion

In conclusion, EPTB was caused exclusively by *M*. *tuberculosis* and the Uganda genotype was the predominant genotype, although at a lower frequency than in studies of PTB in the same location. The spoligotypes were in most cases similar to those causing PTB with the exception of SIT420 which predominated in EPTB but are mainly lacking in other studies of PTB. Exploring the diversity of *M*. *tuberculosis* is essential in appreciating the clinical manifestations and pathogenesis of the disease, as well as the development of new diagnostic strategies.

Mtb lineages are geographically restricted and may adapt to local human populations. Effective vaccine candidates may in future have to be evaluated against prevailing Mtb genotypes and host genetic backgrounds.

## Additional files

Additional file 1:
**Table S1.** A summary of genotyping, demographic and epidemiologic data on 121 *M. tuberculosis* strains isolated from Ugandan patient with tuberculous lymphadenitis. Additional file 2:
**Table S2.** Correlation of the major spoligotypes with HIV status. Additional file 3:
**Table S3a.** Correlation of the various lineages with sex. **Table S3b.** Correlation between the predominant spoligotypes and age.

## Abbreviations

TB: tuberculosis
HIV: human immunodeficiency virus
ZN: Ziehl–Neelsen
M.tb: mycobacteria tuberculosis
MOTT: mycobacteria other than tuberculosis
FNA: fine needle aspiration
MGIT: mycobacterial growth indicator tube
CAS: Central Asian Strain
EPTB: extrapulmonary TB
DR: direct repeat locus
PCR: polymerase chain reaction
SIT: spoligotype international type
