# Supplementary material for: Predominance of Uganda genotype of Mycobacterium tuberculosis isolated from Ugandan patients with tuberculous lymphadenitis
Source: BMC Res Notes. 2015 Sep 1;8:398. doi: 10.1186/s13104-015-1362-y (PMC4556223; doi:10.1186/s13104-015-1362-y)
Supplement: Additional file 1: — Table S1. A summary of genotyping, demographic and epidemiologic data on 121 M. tuberculosis strains isolated from Ugandan patient with tuberculous lymphadenitis. [file 13104_2015_1362_MOESM1_ESM.pdf]

Supplemental Table S1: A summary of genotyping, demographic and epidemiologic data on 121 *M. tuberculosis*strains isolated from Ugandan patient with tuberculous lymphadenitis.

IsoNumber	Strain	Year	Sex	Age	Spoligotype Description	Octal code	SIT	Lineage	RD724 deletion
UGA0420110w10167	w10167	2011	F	54	□□□□□□□□□□□□□□□□□□□□□□□□□□□□□□□□□□■■■■■■■■■	000000000003771	1	Beijing	No
UGA0420110w10627	w10627	2011	M	32	□□□□□□□□□□□□□□□□□□□□□□□□□□□□□□□□□□■■■■■■■■■	000000000003771	1	Beijing	No
UGA0420110w11833	w11833	2011	M	22	□□□□□□□□□□□□□□□□□□□□□□□□□□□□□□□□□□■■■■■■■■■	000000000003771	1	Beijing	No
UGA0420110w11826	w11826	2011	M	28	□□□□□□□□□□□□□□□□□□□□□□□□■■■■■■■■□□□□■■■■■■■	000000007760771	4	Unknown	No
UGA0420110w5908	w5908	2011	M	52	□□□□□□□□□□□□□□□□□□□□□□□□■■■■■■■■□□□□■■■■■■■	000000007760771	4	Unknown	No
UGA0420110w7682	w7682	2011	M	26	□□□□□□□□□□□□□□□□□□□□□□□□■■■■■■■■□□□□■■■■■■■	000000007760771	4	Unknown	No
UGA0420110W11123	W11123	2011	F	33	■■■□□□□■■□■■■■■■■■■□□□□□□□□□□□□□□□□■■■■■■■■	703377400001771	21	CAS1-Kili	No
UGA0420110W3267	W3267	2011	M	24	■■■□□□□■■□■■■■■■■■■□□□□□□□□□□□□□□□□■■■■■■■■	703377400001771	21	CAS1-Kili	No
UGA0420110w4932	w4932	2011	M	25	■■■□□□□■■□■■■■■■■■■□□□□□□□□□□□□□□□□■■■■■■■■	703377400001771	21	CAS1-Kili	No
UGA0420110w8133	w8133	2011	M	27	■■■□□□□■■□■■■■■■■■■□□□□□□□□□□□□□□□□■■■■■■■■	703377400001771	21	CAS1-Kili	No
UGA0420110w9133	w9133	2011	M	30	■■■□□□□■■□■■■■■■■■■□□□□□□□□□□□□□□□□■■■■■■■■	703377400001771	21	CAS1-Kili	No
UGA0420110w5012	w5012	2011	F	30	■■■□□□□■■■■■■■■■■■■■■■□□□□□□□□□□□□■■□□■■■■■	703777740003171	25	CAS1-Delhi	No
UGA0420110w11831	w11831	2011	M	18	■■■□□□□■■■■■■■■■■■■■■■□□□□□□□□□□□□■■■■■■■■■	703777740003771	26	CAS1-Delhi	No
UGA0420110w7960	w7960	2011	M	22	■■■□□□□■■■■■■■■■■■■■■■□□□□□□□□□□□□■■■■■■■■■	703777740003771	26	CAS1-Delhi	No
UGA0420110w9868	w9868	2011	M	49	■■■□□□□■■■■■■■■■■■■■■■□□□□□□□□□□□□■■■■■■■■■	703777740003771	26	CAS1-Delhi	No
UGA0420110w11642	w11642	2011	M	32	■■■■■■■■■■■■■■■■■■■■■■■■■■■■■■■■□□□□■■■□■■■	777777777760731	52	T2	Yes
UGA0420110w3357	w3357	2011	F	32	■■■■■■■■■■■■■■■■■■■■■■■■■■■■■■■■□□□□■■■□■■■	777777777760731	52	T2	Yes
UGA0420110w3494	w3494	2011	-	-	■■■■■■■■■■■■■■■■■■■■■■■■■■■■■■■■□□□□■■■□■■■	777777777760731	52	T2	Yes
UGA0420110w9592	w9592	2011	F	15	■■■■■■■■■■■■■■■■■■■■■■■■■■■■■■■■□□□□■■■□■■■	777777777760731	52	T2	Yes
UGA0420110w10439	w10439	2011	M	60	■■■■■■■■■■■■■■■■■■■■■■■■■■■■■■■■□□□□■■■■■■■	777777777760771	53	T1	No
UGA0420110w11522	w11522	2011	M	33	■■■■■■■■■■■■■■■■■■■■■■■■■■■■■■■■□□□□■■■■■■■	777777777760771	53	T1	No
UGA0420110w3248	w3248	2011	F	24	■■■■■■■■■■■■■■■■■■■■■■■■■■■■■■■■□□□□■■■■■■■	777777777760771	53	T1	No
UGA0420110w5187	w5187	2011	M	35	■■■■■■■■■■■■■■■■■■■■■■■■■■■■■■■■□□□□■■■■■■■	777777777760771	53	T1	No
UGA0420110w5252	w5252	2011	F	30	■■■■■■■■■■■■■■■■■■■■■■■■■■■■■■■■□□□□■■■■■■■	777777777760771	53	T1	No
UGA0420110w7939	w7939	2011	M	30	■■■■■■■■■■■■■■■■■■■■■■■■■■■■■■■■□□□□■■■■■■■	777777777760771	53	T1	No
UGA0420110w8153	w8153	2011	M	27	■■■■■■■■■■■■■■■■■■■■■■■■■■■■■■■■□□□□■■■■■■■	777777777760771	53	T1	No
UGA0420110w9505	w9505	2011	M	49	■■■■■■■■■■■■■■■■■■■■■■■■■■■■■■■■□□□□■■■■■■■	777777777760771	53	T1	No
UGA0420110w9960	w9960	2011	M	28	■■■■■■■■■■■■■■■■■■■■■■■■■■■■■■■■□□□□■■■■■■■	777777777760771	53	T1	No
UGA0420110w9294	w9294	2011	M	22	■■■■■■■■■■■■■■■■■■■■■■■■■■■■■■■■□□□□■■■■■■■	777777777760771	53	T1	No
UGA0420110w7920	w7920	2011	F	31	■■■■■■■■■■■■■■■■■■■■■■■■■■■■■■■■□□□□■■■■■■■	777777777760771	53	T1	No
UGA0420110w5440	w5440	2011	M	30	■■■■■■■■■■■■■■■■■■■■■■■■■■■■■■■■□□■■■■■■■■■	777777777763771	54	Manu2	No
UGA0420110w11858	w11858	2011	F	47	■■■■■■■■■■■■■■■■■■■■□□□□■■□□□□■■□□□□■■■■■■■	777777606060771	59	LAM11-ZWE	No
UGA0420110w12135	w12135	2011	F	29	■■■■■■■■■■■■■■■■■■■■□□□□■■□□□□■■□□□□■■■■■■■	777777606060771	59	LAM11-ZWE	No
UGA0420110w7401	w7401	2011	M	26	■■■■■■■■■■■■■■■■■■■■□□□□■■□□□□■■□□□□■■■■■■■	777777606060771	59	LAM11-ZWE	No
UGA0420110w10691	w10691	2011	F	28	■■■■■■■■■■■■■■■■■■■■■■■■■■■■■■■■□□□□■■■□□■■	777777777760711	78	T	No
UGA0420110w7275	w7275	2011	F	50	■■■■■■■■■■■■■■■■■■■■■■■■■■■■■■■■□□□□■■■□□■■	777777777760711	78	T	No
UGA0420110w10884	w10884	2011	F	35	□□□□□□□□□□□□□□□□□□□□□□□□■■■■■■■■□□□□■■■□■■■	000000007760731	125	T2/LAM3 (controversial signature)	Yes
UGA0420110w11161	w11161	2011	F	38	□□□□□□□□□□□□□□□□□□□□□□□□■■■■■■■■□□□□■■■□■■■	000000007760731	125	T2/LAM3 (controversial signature)	Yes

UGA0420110w11824	w11824	2011	F	30	■□□■■■■■■■■■■■■■■■■■■■■■■■■■□□□□■□■■■■■■■■■	477777777413771	126	EAI5	No
UGA0420110w4902	w4902	2011	F	29	■□□■■■■■■■■■■■■■■■■■■■■■■■■■□□□□■□■■■■■■■■■	477777777413771	126	EAI5	No
UGA0420110w4919	w4919	2011	M	27	■□□■■■■■■■■■■■■■■■■■■■■■■■■■□□□□■□■■■■■■■■■	477777777413771	126	EAI5	No
UGA0420110w3265	w3265	2011	M	20	■■□□■■■■■■■■■■■■□■■■■■■■■■■■■■■□□□□■■■□■■□	637775777760730	128	T2-Uganda	Yes
UGA0420110w3601	w3601	2011	M	32	■■□□■■■■■■■■■■■■□■■■■■■■■■■■■■■□□□□■■■□■■□	637775777760730	128	T2-Uganda	Yes
UGA0420110w3833	w3833	2011	F	25	■■□□■■■■■■■■■■■■□■■■■■■■■■■■■■■□□□□■■■□■■□	637775777760730	128	T2-Uganda	Yes
UGA0420110w5083	w5083	2011	M	-	■■□□■■■■■■■■■■■■□■■■■■■■■■■■■■■□□□□■■■□■■□	637775777760730	128	T2-Uganda	Yes
UGA0420110w5588	w5588	2011	F	27	■■□□■■■■■■■■■■■■□■■■■■■■■■■■■■■□□□□■■■□■■□	637775777760730	128	T2-Uganda	Yes
UGA0420110w7222	w7222	2011	M	33	■■□□■■■■■■■■■■■■□■■■■■■■■■■■■■■□□□□■■■□■■□	637775777760730	128	T2-Uganda	Yes
UGA0420110w8897	w8897	2011	M	3	■■□□■■■■■■■■■■■■□■■■■■■■■■■■■■■□□□□■■■□■■□	637775777760730	128	T2-Uganda	Yes
UGA0420110w3646	w3646	2011	M	20	■■□□■■■■■■■■■■■■□■■■■■■■■■■■■■■□□□□■■■□■■□	637775777760730	128	T2-Uganda	Yes
UGA0420110w2981	w2981	2011	-	-	■■□□■■■■■■■■■■■■□■■■■■■■■■■■■■■□□□□■■■□■■□	637775777760730	128	T2-Uganda	Yes
UGA0420110w7850	w7850	2011	F	15	■■■■■■■■■■■■■■■■■■■■■■■■■■■■■■■■□□□□■■■□■■□	777777777760730	135	T2-Uganda	Yes
UGA0420110w10115	w10115	2011	F	15	■■■■■■■■■■■■■■■■■■■■■■■■■■■■■■■■□□□□■■■□■■□	777777777760730	135	T2-Uganda	Yes
UGA0420110w11166	w11166	2011	F	38	■■■■■■■■■■■■■■■■■■■■■■■■■■■■■■■■□□□□■■■□■■□	777777777760730	135	T2-Uganda	Yes
UGA0420110w11713	w11713	2011	F	24	■■■■■■■■■■■■■■■■■■■■■■■■■■■■■■■■□□□□■■■□■■□	777777777760730	135	T2-Uganda	Yes
UGA0420110w11761	w11761	2011	F	46	■■■■■■■■■■■■■■■■■■■■■■■■■■■■■■■■□□□□■■■□■■□	777777777760730	135	T2-Uganda	Yes
UGA0420110w12243	w12243	2011	M	32	■■■■■■■■■■■■■■■■■■■■■■■■■■■■■■■■□□□□■■■□■■□	777777777760730	135	T2-Uganda	Yes
UGA0420110w3160	w3160	2011	F	27	■■■■■■■■■■■■■■■■■■■■■■■■■■■■■■■■□□□□■■■□■■□	777777777760730	135	T2-Uganda	Yes
UGA0420110w4792	w4792	2011	M	61	■■■■■■■■■■■■■■■■■■■■■■■■■■■■■■■■□□□□■■■□■■□	777777777760730	135	T2-Uganda	Yes
UGA0420110w4889	w4889	2011	M	22	■■■■■■■■■■■■■■■■■■■■■■■■■■■■■■■■□□□□■■■□■■□	777777777760730	135	T2-Uganda	Yes
UGA0420110w8619	w8619	2011	F	29	■■■■■■■■■■■■■■■■■■■■■■■■■■■■■■■■□□□□■■■□■■□	777777777760730	135	T2-Uganda	Yes
UGA0420110w11832	w11832	2011	F	32	■■■■■■■■■□□□□□□□□□□■■■■■■■■■■■■■□□□□■■■■■■■	777000377760771	149	T3-ETH	No
UGA0420110w11834	w11834	2011	F	28	■■■■■■■■■■■■■■■■■■□□□□□□■□□□□□□■□□□□■■■■■■■	777777004020771	182	H1	No
UGA0420110w3204	w3204	2011	F	39	■■■□■■■■■■■■■■■■■■■■■■■■■■■■■■■■□□□□■■■■■■■	737777777760771	205	T1	No
UGA0420110w7209	w7209	2011	F	20	■■■■■■■■■■■■■■■■■□■■■■■■■■■■■■■■□□■■■■■■■■■	777776777763771	226	Manu2	No
UGA0420110w9080	w9080	2011	F	29	■■■□□□□□□□■■■■■■■■■■■■□□□□□□□□□□□□■■■■■■■■■	700377740003771	288	CAS2	No
UGA0420110w11418	w11418	2011	F	52	■■■□□□□■■■■■■■■■■■■■□□□□□□□□□□□□□□□■■■■■■■■	703777600001771	356	CAS1-Delhi	No
UGA0420110w3433	w3433	2011	F	26	■■■□□□□■■■■■■■■■■■■■□□□□□□□□□□□□□□□■■■■■■■■	703777600001771	356	CAS1-Delhi	No
UGA0420110w4731	w4731	2011	F	22	■■■□□□□■■■■■■■■■■■■■□□□□□□□□□□□□□□□■■■■■■■■	703777600001771	356	CAS1-Delhi	No
UGA0420110w5186	w5186	2011	F	21	■■■□□□□■■■■■■■■■■■■■□□□□□□□□□□□□□□□■■■■■■■■	703777600001771	356	CAS1-Delhi	No
UGA0420110w9463	w9463	2011	F	17	■■■□□□□■■■■■■■■■■■■■□□□□□□□□□□□□□□□■■■■■■■■	703777600001771	356	CAS1-Delhi	No
UGA0420110w12337	w12337	2011	F	27	■■■□□■■■■■■■■■■■■■■■■■■■■■■■■■■■□□□□■■■■■■■	717777777760771	358	T1	No
UGA0420110w10948	w10948	2011	F	8	■■□□■■■■■■■■■■■■□□■■■■■■■■■■■■■■□□□□■■■□■■□	637774777760730	420	T2-Uganda	Yes
UGA0420110w11033	w11033	2011	M	34	■■□□■■■■■■■■■■■■□□■■■■■■■■■■■■■■□□□□■■■□■■□	637774777760730	420	T2-Uganda	Yes
UGA0420110w11079	w11079	2011	F	28	■■□□■■■■■■■■■■■■□□■■■■■■■■■■■■■■□□□□■■■□■■□	637774777760730	420	T2-Uganda	Yes
UGA0420110w11510	w11510	2011	F	28	■■□□■■■■■■■■■■■■□□■■■■■■■■■■■■■■□□□□■■■□■■□	637774777760730	420	T2-Uganda	Yes
UGA0420110w12241	w12241	2011	F	7	■■□□■■■■■■■■■■■■□□■■■■■■■■■■■■■■□□□□■■■□■■□	637774777760730	420	T2-Uganda	Yes
UGA0420110w2639	w2639	2011	M	24	■■□□■■■■■■■■■■■■□□■■■■■■■■■■■■■■□□□□■■■□■■□	637774777760730	420	T2-Uganda	Yes
UGA0420110w3461	w3461	2011	M	25	■■□□■■■■■■■■■■■■□□■■■■■■■■■■■■■■□□□□■■■□■■□	637774777760730	420	T2-Uganda	Yes
UGA0420110w5422	w5422	2011	F	20	■■□□■■■■■■■■■■■■□□■■■■■■■■■■■■■■□□□□■■■□■■□	637774777760730	420	T2-Uganda	Yes
UGA0420110w5596	w5596	2011	F	29	■■□□■■■■■■■■■■■■□□■■■■■■■■■■■■■■□□□□■■■□■■□	637774777760730	420	T2-Uganda	Yes
UGA0420110w6209	w6209	2011	F	26	■■□□■■■■■■■■■■■■□□■■■■■■■■■■■■■■□□□□■■■□■■□	637774777760730	420	T2-Uganda	Yes
UGA0420110w7937	w7937	2011	M	32	■■□□■■■■■■■■■■■■□□■■■■■■■■■■■■■■□□□□■■■□■■□	637774777760730	420	T2-Uganda	Yes

UGA0420110w8041	w8041	2011	M	32	■■□□■■■■■■■■■■■■□□■■■■■■■■■■■■■■□□□□■■■□■■□	637774777760730	420	T2-Uganda	Yes
UGA0420110w9727	w9727	2011	F	22	■■□□■■■■■■■■■■■■□□■■■■■■■■■■■■■■□□□□■■■□■■□	637774777760730	420	T2-Uganda	Yes
UGA0420110w11154	w11154	2011	M	18	■■■■■■■■■■■■■■■■■■■■■■■■■■■■■■■□□□□■■■■■■□	777777777760770	522	T1	No
UGA0420110w8025	w8025	2011	F	30	■■■■■■■■■■■■■■■■■■■■■■■■■■■■■■■□□□□■■■■■■□	777777777760770	522	T1	No
UGA0420110w12302	w12302	2011	F	44	■■■	777777777777771	523	Manu_ancestor	No
UGA0420110w11703	w11703	2011	F	34	■■■■■■■■■■■■■■■■■■■□□□□□□■■■■■■■□□□□■■■□■■□	777777403760730	590	T2-Uganda	Yes
UGA0420110w11939	w11939	2011	F	31	■■■■■■■■■■■■■■■■■■■□□□□□□■■■■■■■□□□□■■■□■■□	777777403760730	590	T2-Uganda	Yes
UGA0420110w5254	w5254	2011	M	34	■■■■■■■■■■■■■■■■■■■□□□□□□■■■■■■■□□□□■■■□■■□	777777403760730	590	T2-Uganda	Yes
UGA0420110w9992	w9992	2011	M	30	■■■■■■■■■■■■■■■■■■■□□□□□□■■■■■■■□□□□■■■□■■□	777777403760730	590	T2-Uganda	Yes
UGA0420110w5554	w5554	2011	M	41	■■■■■■■■■□■■■■■■■■■■■■■■■■■■■■■■□□□□■■■■■■■	777377777760771	1166	T1	No
UGA0420110w11623	w11623	2011	M	29	■■□■■■■■■■■■□■■■■■■■■■■■■■■■■■■■□□□□■■■□■■■	677737777760731	1339	T	Yes
UGA0420110w5757	w5757	2011	M	38	■■■■■■■■■■■■■□□□□□□□□□□□□□□□□□□□□□□□□□□□□□□	777740000000000	1570	Unknown	No
UGA0420110Pat 5.10	Pat 5.10	2011	F	35	■■■■■■■■■□■■■■■■■■■■■■■■■■■■■■■■□□□□■■■□■■■	777377777760731	2188	T2	Yes
UGA0420110w9726	w9726	2011	M	-	■■■■■■■■■■■■■■■■■■■■■■■■■□■■■■■■□□□□■■■□■■■	777777775760731	2867	T2	Yes
UGA0420110w9869	w9869	2011	F	17	■■■■■■■■■■■■■■■■■■■■■■■■■□■■■■■■□□□□■■■□■■■	777777775760731	2867	T2	Yes
UGA0420110w7787	w7787	2011	M	32	□□□□□□□■■■■■■■■■■■■■■■□□□□□□□□□□□□■□■■■■■■■	003777740002771	3502	Unknown	No
UGA0420110w9958	w9958	2011	M	32	■■■■■■■■■■■■■■■■■■■■■■■■■■■■■■■□■□□□■■■■■■■	777777777750771	4008	Manu3	No
UGA0420110w11521	w11521	2011	M	27	■■■■■■■■■□■■■□■■■■■■■■■■■■■■□■■■■■■□□□■■■□■■■	777357776770731	4059*	Manu3	No
UGA0420110w11550	w11550	2011	F	4	■■■■■■■■■□■■■□■■■■■■■■■■■■■■□■■■■■■□□□■■■□■■■	777357776770731	4059*	Manu3	No
UGA0420110w10508	w10508	2011	F	26	□□■■■■■■■■■■■■■■■■■■■■■■■■■■■■■■□□□□■■□□□□■	177777777760601	4060*	T1	No
UGA0420110w11624	w11624	2011	M	11	■■■■■■■■■■■■■■■■□■■■■■■■■■■■■■■■□□□□■■■□■■□	777775777760730	4061*	T2-Uganda	Yes
UGA0420110w11823	w11823	2011	F	30	■■■■■■■■■■■■■■■■□■■■■■■■■■■■■■■■□□□□■■■□■■□	777775777760730	4061*	T2-Uganda	Yes
UGA0420110w11655	w11655	2011	M	43	□■□□■■■■■■■■■■■■□■■■■■■■■■■■■■■■□□□□■■■□■■□	237775777760730	4062*	T2-Uganda	Yes
UGA0420110w11766	w11766	2011	M	37	■■■■■■■■■■■■■■□□□■□□□□□□■■■■■■■■□□□□■■□■■□■	777761007760661	Or01	LAM9	No
UGA0420110w8778	w8778	2011	M	40	□□□■■■□■■■■■■■■■■■■■■■■■■■■■□□□□■□■■□□□■■■■	073777777413071	Or02	EAI3-IND	No
UGA0420110w5688	w5688	2011	F	12	■■■□□□□■■□■■■■■■■■■■■■■□□□□□□□□□□□□■□■■□■■■■	703377740002671	Or03	CAS1-Delhi	No
UGA0420110w11050	w11050	2011	M	32	■■■□□■■■■■■■■■■■■□■■■■□□□□□□□□□□□□□□□□■□■■□	717776740000130	Or04	Unknown	No
UGA0420110w12134	w12134	2011	F	42	■■■■■■■■■□■■■■■■■■■■□■■■■■■■■■■■□□□□■■□■■■■	777377677760671	Or05	T1	No
UGA0420110w11723	w11723	2011	F	13	■■■□□■□■■□■■■■■■■■■■■■■■■■■■■■■■□□□■■■■■■■■	713377777761771	Or06	Unknown	No
UGA0420110w12036	w12036	2011	F	23	■■■□□■□■■□■■■■■■■■■■■■■■■■■■■■■■□□□□■■■■■■■	713377777760771	Or07	T1	No
UGA0420110w9132	w9132	2011	F	3	■■■■■■■■■■■■□■■■□□■■■■■■■■■■■■■■□□□□■■■□■■□	777734777760730	Or08	T2-Uganda	Yes
UGA0420110w11425	w11425	2011	M	30	■■□■■■■■■■■■□■■■■■■■■■■■■■■■■■■■□□□□■■■□■■□	677737777760730	Or09	T2-Uganda	Yes
UGA0420110w5791	w5791	2011	M	30	■■■■■■■■■■□■■■□■□□■■■■■■■■■■■■■■□□□□■■■□■■□	777564777760730	Or10	T2-Uganda	Yes
UGA0420110w10349	w10349	2011	M	28	■■■■■■□■■■■■■■■■□□■■■■■■■■■■■□□■□□□□■■■□■■□	773774777620730	Or11	H3	No
UGA0420110w7683	w7683	2011	F	31	□■□□■■■■■■■■■■■■□□■■■■■■■■■■■■■■□□□□■■■□■■□	237774777760730	Or12	T2-Uganda	Yes
UGA0420110w8168	w8168	2011	F	27	■■□□■■■■■■■■■■■■□□■■■■■■□□■■■■■■□□□□■■■□■■□	637774771760730	Or13	T2-Uganda	Yes
UGA0420110w2638	w2638	2011	F	50	■■□□■□■□■■■■■■■■□□■■■■■■■■■■■■■■□□□□■■■□■■□	625774777760730	Or14	T2-Uganda	Yes
UGA0420110w11021	w11021	2011	F	22	■■■□■■■■■■■■■■■■■■■□□□□□□■■□□■■■□□□□■■■□■■□	737777403160730	Or15	T2-Uganda	Yes
UGA0420110w5758	w5758	2011	M	38	□□□□□□□□□□□□□□□□□□□□□□□□■■■■■□■■□□□□■■■□■■■	000000007660731	Or16	LAM3	No

*Newly created SITs are followed by an asterisk, and orphans spoligotypes are labeled "Or"
