# Supplementary material for: Predominance of Uganda genotype of Mycobacterium tuberculosis isolated from Ugandan patients with tuberculous lymphadenitis
Source: BMC Res Notes. 2015 Sep 1;8:398. doi: 10.1186/s13104-015-1362-y (PMC4556223; doi:10.1186/s13104-015-1362-y)
Supplement: Additional file 2: — Table S2. Correlation of the major spoligotypes with HIV status. [file 13104_2015_1362_MOESM2_ESM.docx]

Supplemental Table S2. Correlation of the Major Spoligotypes with HIV status.

| Spoligotype | HIV (-ve) | HIV (+ve) | P-value |
| --- | --- | --- | --- |
| 490T2 | 8/74 (10.8%) | 4/38 (10.5%) | 1.000 |
| 135T2 | 6/74 (8.1%) | 4/38 (10.5%) | 0.7318 |
| 53T1 | 9/74 (12.2%) | 1/38 (2.6%) | 0.1600 |
