# Supplementary material for: Predominance of Uganda genotype of Mycobacterium tuberculosis isolated from Ugandan patients with tuberculous lymphadenitis
Source: BMC Res Notes. 2015 Sep 1;8:398. doi: 10.1186/s13104-015-1362-y (PMC4556223; doi:10.1186/s13104-015-1362-y)
Supplement: Additional file 3: — Table S3a. Correlation of the various lineages with sex. Table S3b. Correlation between the predominant spoligotypes and age. [file 13104_2015_1362_MOESM3_ESM.docx]

**Supplemental Table S3a**. Correlation of the various lineages with sex.

| SIT Spoligotype |  | Males (58) | | Females (62) | | *P value |
| --- | --- | --- | --- | --- | --- | --- |
|  | Total count | No. | % | No. | % |  |
| 1 Beijing | 3 | 2 | 3 | 1 | 2 | 1.00 |
| 21 CAS-Kili | 5 | 4 | 7 | 1 | 2 | 0.207 |
| 26 CAS1-Dehli | 3 | 3 | 5 | 0 | 0 | 0.110 |
| 52 T2 | 3 | 1 | 2 | 2 | 3 | 1.00 |
| 53 T1 | 11 | 8 | 14 | 3 | 5 | 0.206 |
| 59 LAM11-ZWE | 3 | 1 | 2 | 2 | 3 | 1.00 |
| 78 T | 2 | 0 | 0 | 2 | 3 | 0.497 |
| 125 T2/LAM | 2 | 0 | 0 | 2 | 3 | 0.497 |
| 126 EAI5 | 3 | 1 | 2 | 2 | 3 | 1.00 |
| 128 T2-Uganda | 8 | 6 | 10 | 2 | 3 | 0.273 |
| 135 T2-Uganda | 10 | 3 | 5 | 7 | 11 | 0.767 |
| 356 CAS1-Dehli | 5 | 0 | 0 | 5 | 8 | 0.061 |
| 420 T2-Uganda | 13 | 5 | 9 | 8 | 13 | 0.569 |
| 522 T1 | 2 | 1 | 2 | 1 | 2 | 1.00 |
| 590 T2 | 4 | 2 | 3 | 2 | 3 | 1.00 |
|  |  |  |  |  |  |  |

*Fisher-exact test

Note: The sex of one of the patients with SIT52 was not captured during sample collection.

**Supplemental Table S3b.** Correlation between the predominant spoligotypes and age

| SIT53 vs SIT 420 | SIT 53 | SIT420 | *P-Value |
| --- | --- | --- | --- |
| **Age**  Mean | 30 (SD 8) | 26 (SD 11) | 0.375 |
|  | | | |
| SIT128 vs SIT 135 | SIT 128 | SIT 135 | *P-Value |
| **Age**  Mean | 23(SD 10) | 31(SD14) | 0.401 |

*Independent-Samples T Test
